# Novel off-Targeting Events Identified after Genome Wide Analysis of CRISPR-Cas Edited Pigs

**DOI:** 10.1089/crispr.2024.0012

**Published:** 2024-06-21

**Authors:** Bethany K. Redel, Junchul Yoon, Emily Reese, Hong An, Kyungjun Uh, Paula R. Chen, Randall S. Prather, Kiho Lee

**Affiliations:** ^1^ USDA-ARS, Plant Genetics Research Unit, Columbia, Missouri, USA.; ^2^ Division of Animal Sciences, University of Missouri, Columbia, Missouri, USA.; ^3^ Bioinformatics and Analytics Core, University of Missouri, Columbia, Missouri, USA.; ^4^ Futuristic Animal Resource & Research Center (FARRC), Korea Research Institute of Bioscience and Biotechnology (KRIBB), Chungcheongbuk-do, Republic of Korea.; ^5^ National Swine Resource and Research Center, Columbia, Missouri, USA.

## Abstract

CRISPR-Cas technology has transformed our ability to introduce targeted modifications, allowing unconventional animal models such as pigs to model human diseases and improve its value for food production. The main concern with using the technology is the possibility of introducing unwanted modifications in the genome. In this study, we illustrate a pipeline to comprehensively identify off-targeting events on a global scale in the genome of three different gene-edited pig models. Whole genome sequencing paired with an off-targeting prediction software tool filtered off-targeting events amongst natural variations present in gene-edited pigs. This pipeline confirmed two known off-targeting events in *IGH* knockout pigs, *AR* and *RBFOX1*, and identified other presumably off-targeted loci. Independent validation of the off-targeting events using other gene-edited DNA confirmed two novel off-targeting events in *RAG2/IL2RG* knockout pig models. This unique strategy offers a novel tool to detect off-targeting events in genetically heterogeneous species after genome editing.

## Introduction

The ability to introduce site specific modifications into the pig genome using the clustered, regularly interspaced, short palindromic repeat (CRISPR) technology has revolutionized the production of gene-edited (GE) pigs for both agriculture and biomedical applications. Advancements in this technology have paved the way for novel methods to produce GE pigs at high efficiencies. While the benefit of the technology is evident, the possibility of causing unintended modifications elsewhere in the genome has remained the main concern. The CRISPR system often utilizes targeted genomic double strand breaks (DSBs), produced by the CRISPR associated protein 9 (Cas9), to introduce site-specific genetic modifications.^1^ A 20-nucleotide single guide RNA (sgRNA) directs the Cas9 nuclease to a specific location in the genome, and Cas9 produces DSBs if a protospacer adjacent motif (PAM) containing an NGG site is present.^2^ The DSBs activate the cell’s DNA repair mechanism; however, small changes to the genomic sequences, that is, insertions-deletions (InDels), can be introduced as an outcome. Since the DSB relies on the short 20 nucleotide guide sequence to direct the endonuclease, Cas9, unintended modifications, known as off-targeting events, to the genome have been considered to be a potential side effect of the technology. In addition, if utilized in embryos, there is a chance for a high rate of mosaicism to occur.^3^ This creates a challenge when studying founder animals that may contain more than two alleles, confounding phenotyping data.

Pigs are an ideal animal model for human diseases^4^ and are the second most consumed meat globally.^5^ Therefore, the use of gene editing technology in pigs for promoting human health research and identifying ways to improve pig production for agriculture purposes has increased immensely.^6^ Unlike the genome of traditional laboratory animals such as the mouse, the pig is generally not inbred, and therefore, a high level of natural variation exists in the genome among different breeds. The high level of natural variation markedly complicates the detection of true off-targeting events as there is not an ideal way to filter only the modifications originated from CRISPR-Cas system when natural variation exists against a reference genome. In addition, during GE pig production, unknown genetic background is often introduced maternally as oocytes are provided by abattoir-derived ovaries, and therefore, the genetic background of founder GE pigs is not fully characterized. Various prediction software and biochemical methods have been developed to predict off-targeting events and shown to be effective;^7–9^ however, to the best of our knowledge, there is not a system that can rapidly identify off-targeting events in gene-edited organisms carrying unknown genetic background. As the CRISPR-Cas system is now routinely used to introduce targeted modifications into nonconventional animal models such as pigs, which carry a heterogeneous genome and the parental genetic information is not necessarily available, it is important to develop a strategy that allows us to identify potential off-targeting events. Previously, we detected off-targeting events in gene-edited pigs by searching for targeted loci.^10^ While screening a handful of genes by targeted PCR has been informative, our priority is to identify and screen novel off-targeting events on a global level.

In this study, we utilized whole genome sequencing (WGS) paired with an off-targeting prediction software to evaluate the level of off-targeting events in the genome of three GE pig models carrying different levels of off-targeting events. The system can confirm known off-targeting events in the gene-edited pigs while also detecting novel off-targeting events, thus building an effective approach to overcome the difficulty in screening off-targeting events in heterogeneous animal models.

## Methods

### sgRNA design

**FIG. 1.**
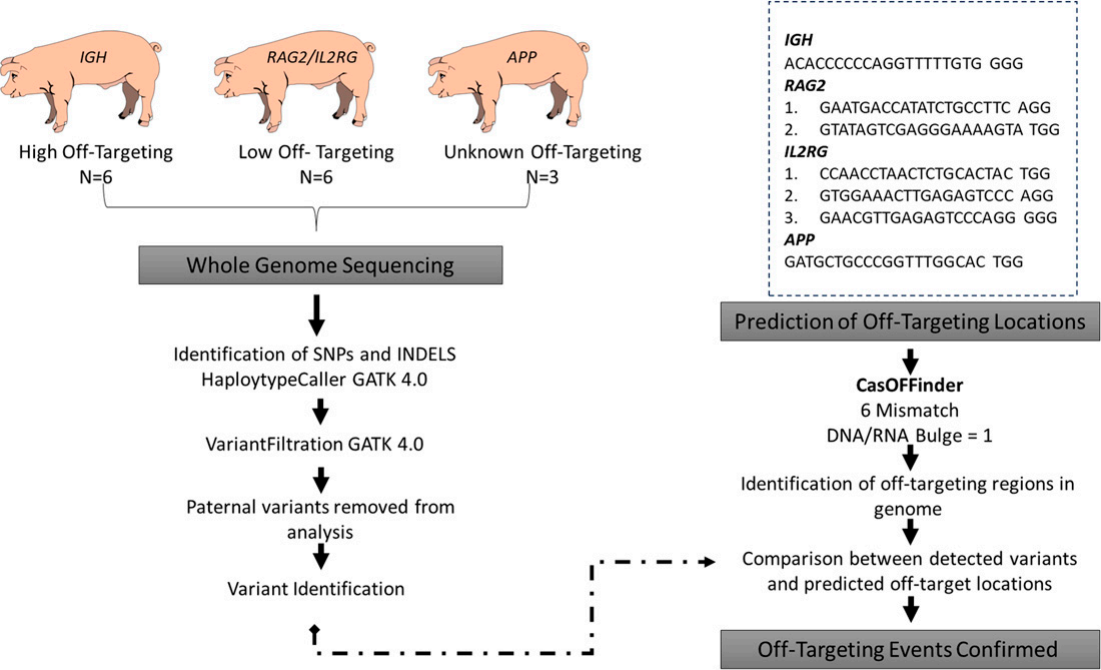
WGS and prediction workflow of identifying off-target modifications in GE pigs.

Design of single guide RNAs (sgRNAs) to generate pigs lacking functional immunoglobulin heavy locus (*IGH*) and carrying modified recombination activating 2 (*RAG2)* and interleukin 2 receptor subunit gamma (*IL2RG)* was previously published.^11,12^ The sgRNAs targeting amyloid beta precursor protein (*APP*) were designed using CRISPOR^13^ as previously described^10^ (Fig. 1). Potential sgRNAs were aligned to the *Sus Scrofa* 11.1 reference genome using the Basic Local Alignment Search Tool (BLAST) to determine specificity. The designed sgRNAs were then *in vitro* transcribed to produce RNA that was coinjected with Cas9 RNA into zygotes as previously described.^14^

### GE embryo and pig production

All animals were maintained according to the approved protocol and standard operating procedures by the Institutional Animal Care and Use Committee of the University of Missouri. Mature oocytes were coincubated with wild-type boar semen for 4 h at 38.5°C in an atmosphere of 5% CO_2_ in air as previously described.^15^ Mature oocytes were fertilized and then injected with 10 ng/µl *APP* sgRNA + 20 ng/µl Cas9 RNA and then incubated in PZM3-MU3 medium^16–18^ at 38.5°C in an atmosphere of 5% O_2_, 90% N_2_, 5% CO_2_ in air until day 6 postfertilization. Individual blastocysts were screened by PCR to determine whether the sgRNA was producing high rates of on-target modifications. Once efficiency was validated, 40 to 50 injected blastocysts were transferred to a surrogate on day 5–6 after estrus was detected. The resulting *APP* founder piglets were collected by cesarean section on day 116 of gestation. Multiple tissues were collected from three *APP* knockout pigs to identify on- and off-target events and mosaicism on a global level.

To validate off-targeting events identified in WGS analysis, sgRNAs targeting *IGH* + Cas9 RNA or *RAG2* and *IL2RG* sgRNAs + Cas9 were injected into zygotes as described above. On day 7 after fertilization, at least 20 single embryos for each model were collected to assess off-target modifications using targeted PCR and subsequent Sanger Sequencing. PCR products from selected presumable off-targeting locations were used for cloning to detect all alleles present in the developing embryos. Off-target modifications were then assessed by aligning the sequence to WT and the *SScrofa* 11.1 genome.

### Whole genome sequencing and on- and off-target identification

For all GE pig models, genomic DNA was isolated from knockout founder pigs (*IGH n* =*6*, *RAG2* and *IL2RG n* = *6*, and *APP n* = *3*, from brain, lung, muscle, spleen, and white blood cells [WBCs]) and from the boars that were used to create the GE embryos. These samples were submitted to the University of Missouri DNA Genomics Technology Core for WGS using NovaSeq flow cell technology. Bioinformatic analyses were conducted on the “Lewis” HPC cluster at the University of Missouri. Variant calling was accomplished using a custom Nextflow workflow to highly parallelize the processes.^19^ Reads were mapped to the *Sscrofa11.1* reference genome^20^ using Minimap2.^21^ Next, duplicated reads were marked and removed using MarkDuplicates in GATK (v4.2.6.1), and variants for each sample were called using HaplotypeCaller in GATK (v4.2.6.1).^22^ Finally, hard filters for both single nucleotide polymorphisms (SNPs) and InDels were applied with VariantFiltration in GATK (v4.2.6.1) following GATK best practices,^23^ and only InDels were used for further analyses.

The CRISPR off-target sites at the genome level were estimated by Cas-OFFinder^24^ using the same reference genome and the sgRNA with allowed mismatch number = 6, DNA bulge size =1, and RNA bulge size = 1. Potential off-targeting loci from the Cas-OFFinder were matched to genome locations from the variance identified in the whole-genome sequencing; identities encompassing 50 bp on both sides of the InDel were analyzed to account for larger deletions or insertions to also be identified. To further filter the off-target sites for each sample, the CRISPR edited pig’s parental line was sequenced. The off-target sites for each sample were excluded if they also appeared in its parental line: Pumba was the paternal line for *IGH* and *RAG2*/*IL2RG* GE pigs, while W60 was the paternal line for the *APP* GE pigs. Finally, the remaining candidate off-target sites were identified as the off-target sites for each sample with its specific CRISPR sgRNA.

Candidate off-target sites were further analyzed by aligning each guide to the specific location in the genome of the GE pigs where off-target modifications were predicted to occur. Potential off-targeting loci carrying sequence identity to sgRNA and the presence of a PAM were suspected to be genetic variation, that is, off-targeting, introduced by the CRISPR-Cas system. Suspected off-targeting driven variations were searched on all pig genomic database available on the NCBI to identify if the variation existed in other pig breeds naturally.

## Results

### WGS of GE founder pigs

To investigate whether off-target modifications exist on a global level in GE pigs, we analyzed the genome of 15 founder pigs representing three different pig models. The genomic DNA from *IGH* and *RAG2*/*IL2RG* pig models generated in previous reports^11,12^ and the genomic DNA of *APP* knockout pigs were generated using sgRNAs that presented high editing efficacy (100%). In previous off-target analysis of the *IGH* GE pigs, two off-target genes (*RBFOX1* and *AR*) were found to contain modifications at the frequency of 70% and 80%, respectively. No off-targeting event(s) were identified in the *RAG2*/*IL2RG* pigs from the previous study. To investigate the off-targeting potential of the guides on a global scale, WGS was completed from genomic DNA of the pigs. The WGS output was aligned to the pig reference genome (*Sscrofa* 11.1) and found to have an average of 96% coverage. Using the Genome Analysis Toolkit, regions harboring variations to the *Sscrofa* 11.1 were filtered to generate variant call format (VCF) files. The number of SNPs and InDels from each pig/sample are listed in Table 1. The average number of SNPs identified in our pig models compared to the reference genome ranged from 4,356,183 to 5,277,150. While lower, the number of InDels identified ranged from 1,360,695 to 1,551,824. The level of variation was similar in wild-type genomic DNA paternal line, Pumba, who is the paternal line for both *IGH* and *RAG2*/*IL2RG*, as the GE founder animals; however, the paternal line of *APP*, W60, had fewer total variants than the other pigs (66,654 InDels and 224,654 SNPs). The significantly lower level of variance is presumably due to the presence of Duroc genetic background in W60; *Sscrofa* 11.1 reference genome originated from a Duroc pig.^25,26^ On-target modifications were identified as expected, that is, all on-target modifications matched Sanger sequencing-based genotypes of each founder pig.

**Table 1.** WGS Analysis of gene edited pigs and paternal lines to identify variation compared to *Sscrofa* 11.1 reference genome

Gene target(s)	Pig ID	Tissue/sample	InDels	SNPs	Total WGS base pairs	Depth of Coverage
APP	2–3	Brain	1,469,016	4,643,433	76,695,677,796	31
APP	2–3	Lung	1,447,811	4,603,331	69,591,416,508	28
APP	2–3	Muscle	1,469,074	4,642,891	77,499,764,876	31
APP	2–3	Spleen	1,360,695	4,385,106	60,547,446,064	24
APP	2–4	Brain	1,445,146	4,637,912	64,358,658,582	26
APP	2–4	Lung	1,481,600	4,670,557	85,768,295,658	34
APP	2–4	Muscle	1,434,455	4,544,395	71,438,287,240	29
APP	2–4	Skin	1,473,349	4,670,862	75,722,129,154	30
APP	2–4	Spleen	1,350,005	4,356,183	64,257,157,288	26
APP	2–4	White Blood Cells	1,417,635	4,568,983	59,646,219,778	24
APP	2–5	Brain	1,466,649	4,684,565	66,213,317,592	26
APP	2–5	Lung	1,491,462	4,713,827	81,340,291,024	33
APP	2–5	Muscle	1,468,760	4,665,613	75,621,948,808	30
APP	2–5	Skin	1,464,065	4,654,918	71,029,163,612	28
APP	2–5	Spleen	1,387,880	4,519,839	49,165,117,404	20
APP	2–5	White Blood Cells	1,495,516	4,716,579	87,637,685,624	35
IGH	1–1	Tail	1,474,688	5,020,137	53,627,492,356	21
IGH	1–2	Tail	1,551,824	5,277,150	68,597,128,016	27
IGH	1–3	Tail	1,524,403	5,151,418	60,549,768,746	24
IGH	2–1	Tail	1,510,486	5,116,713	57,030,638,246	23
IGH	2–2	Tail	1,533,883	5,170,660	58,181,687,690	23
IGH	2–3	Tail	1,507,688	4,991,001	65,578,640,432	26
RAG2/IL2RG (SCID)	1484–2	Tail	1,509,774	4,902,331	71,181,260,778	28
RAG2/IL2RG (SCID)	2501–1	Tail	1,517,047	4,920,510	74,011,997,378	30
RAG2/IL2RG (SCID)	2501–4	Tail	1,460,091	4,788,593	60,687,967,872	24
RAG2/IL2RG (SCID)	2501–5	Tail	1,531,101	4,931,008	76,225,116,194	30
RAG2/IL2RG (SCID)	2501–6	Tail	1,534,566	4,978,238	78,983,128,778	32
RAG2/IL2RG (SCID)	2501–7	Tail	1,549,733	4,991,341	91,450,907,046	37
Wild-type Sire RAG2/IL2RG and IGH	Pumba	Semen	1,552,444	5,157,125	80,628,036,708	32
Wild-type Sire APP	W60	Ear	66,654	224,225	122,051,040,738	49

### Off-target prediction

The high level of variants in our founder pigs compared to the reference was expected as pigs are a heterogeneous animal model; however, the level of variations masked off-targeting events from the dataset and was impossible to extract off-targeting information. Therefore, a list of potential off-target sites was generated using the Cas-OFFinder software^24^ using PAM NGG sites and allowing up to 6 mismatches with one RNA and/or DNA bulge compared to the on-target sequence. The WGS variants that were present in the Cas-OFFinder output predicting the off-target location, including 50 bp on either side of the variant location, were selected for further evaluation. There were a similar number of potential off-target locations identified from each sgRNA with an average of 8,608 ± 116 off-target locations (Table 2). However, as expected, the sgRNA designed to target *IGH* had the highest predicted off-target locations (9,071). The *IGH* sgRNA was designed within an exon that was less than 100 bp; thus, the sgRNA was not projected to have high specificity and have the potential to produce off-target modifications.^10^ From the list generated by Cas-OFFinder, the VCF files were filtered to identify regions obtained from Cas-OFFinder and then the sorted variants were evaluated to determine whether they were a result of unintended editing by the CRISPR-Cas system (Fig. 1). Since the genetic background of the paternal genome was known, variations that originated from the paternal genome were also excluded.

**Table 2.** Predicted off-target sites identified by Cas-OFFinder

sgRNA	Cas-OFFinder off-Target Sites
RAG1.2	8857
RAG2.2	8393
IL2RG.1	8120
IL2RG.2	8614
IL2RG.3	8562
IGH	9071
APP	8642

**FIG. 2.**
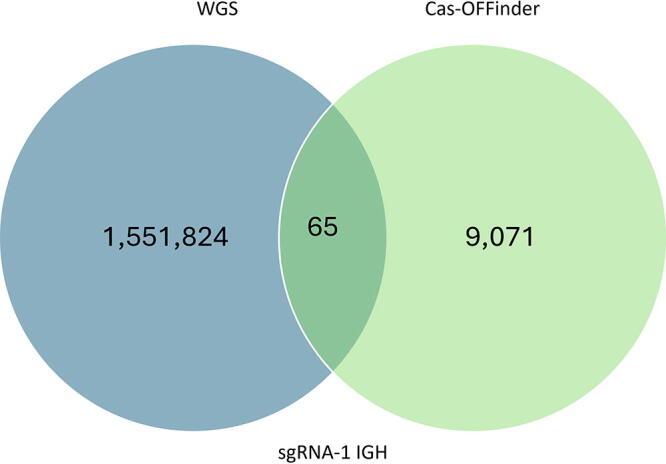
Representative Venn diagram illustrating the number of overlapping off-target locations between WGS and Cas-OFFinder for sgRNA-1 used to create *IGH* 1–2 pig.

This approach significantly reduced the number of potential off-targeting locations. For example, over 1 million locations were found to carry InDel variations against the reference genome (Table 1). By utilizing Cas-OFFinder, potential off-targeting sites were reduced to <100 locations carrying InDels in the six *IGH* knockout pigs (Fig. 2). The sequence identity of the potential off-target locations against sequences of CRISPR-Cas system was verified using the alignment tool in *VectorBuilder* to determine if the variations could be derived from the CRISPR-Cas system (Fig. 2). We analyzed whether there was an adjacent PAM sequence that could induce Cas9 to introduce off-target event(s). The variations detected included on-target allele modifications that were previously confirmed from genotyping; however, in one *APP* GE pig, we identified a different composition of alleles in different tissues, indicating mosaicism in the animal.

### Identification of GE sequence variation of founder pigs

The pipeline confirmed previously detected off-targeting events in *IGH* knockout pigs, that is, *AR* and *RBFOX1*, and generate a list of novel off-target sites in the genome (Table 3). Each location identified, whether in an intron/exon or unannotated, was next to a PAM sequence, which could make them more susceptible to endonuclease activity by Cas9. Two additional prospective off-target locations were identified in *IGH* knockout pigs and eight potential off-targeting locations were discovered in the genome of *RAG2*/*IL2RG* pigs (Table 3). Each sgRNA used to generate *RAG2/IL2RG* pigs may have contributed to introducing 1–4 off-targeting events. For instance, sgRNA designed to target *IL2RG* gene may have introduced an unintended modification to *DDRGK1*; two out of six edited pigs carried variation in the gene compared to the reference sequence.

**Table 3.** List of novel off-target genomic locations identified by screening WGS variants paired with Cas-OFFinder prediction software

Mismatching sgRNA	Gene	Location on chromosome	Intron/Exon	Reference	Number of off-target events
RAG2-1	*HHAT*	NC_010451.4 (132518281-132518381)	Intron	XM_013979981.2	1/6 (16.7%)
RAG2-2	*ECT2L*	NC_010443.5 (25595738-25595838)	Intron	XM_021087372.1	1/6 (16.7%)
RAG2-2	*UBR1*	NC_010443.5 (128356823-128356923)	Intron	XM_021097089	1/6 (16.7%)
RAG2-2	*FAM155A*	NC_010453.5 (74742560-74742660)	Exon	XM_021065383.1	1/6 (16.7%)
RAG2-2	*LOC102159977*	NC_010445.4 (117155315-117155415)	LncRNA	XR_002342830.1	1/6 (16.7%)
IL2RG-1	*LOC110260974*	NC_010448.4 (41622985-41623085)	LncRNA	XR_002344622.1	1/6 (16.7%)
IL2RG-3	*LOC110257355*	NC_010459.5 (40282022-40282122)	LncRNA	XR_002339844.1	1/6 (16.7%)
IL2RG-3	*DDRGK1.1*	NC_010459.5 (32483037-32483139)	Exon	NM_001190212.1	2/6 (33.3%)
IGH-2	*RPS6KC1*	NC_010451.4 (130251247-130251272)	Intron	XM_021063835.1	1/6 (16.7%)
IGH-2	*LOC102163174*	NC_010456.5 (60538300-60538400)	Unannotated	XR_002338974.1	1/6 (16.7%)

### Validation of the off-targeting events in two pig models

To validate whether these identified sites were true off-targeting events, knockout embryos were generated independently by injecting designed CRISPR-Cas system targeting *IGH* or *RAG2*/*IL2RG* into fertilized oocytes. Genotyping of the embryos revealed that most of the potential off-targeting sites were either natural variations existing in other pig breeds or the frequency was too low to detect. For example, two embryos injected with sgRNA for *IGH* contained a 4 bp deletion in the intron of ribosomal protein S6 kinase c1 (*RPS6KC1*) compared to the *Sscrofa* 11.1. However, the variation was also observed in the genome of Ningxiang pig breed, thus determined as a natural variation rather than an off-targeting event by the Cas9 nuclease. Similarly, most of the potential off-targeting locations identified from the WGS were confirmed as natural variations or no off-targeting events were identified from the independent screening.

**FIG. 3.**
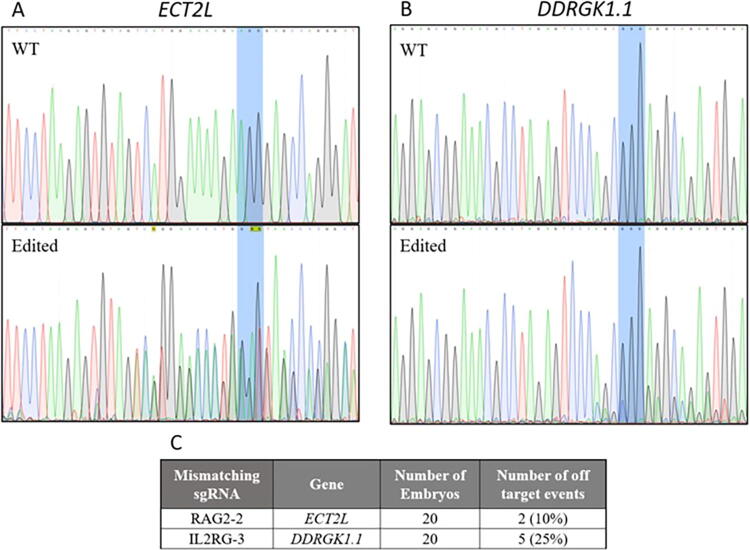
Sanger sequencing of individual blastocysts for novel off-target genes *DDRGK1* and *ECT2L* in *RAG2/IL2RG* sgRNA injected embryos. Representative chromatograms for wild-type control embryos and injected embryos assayed for *ECT2*L **(A)** and *DDRGK1* **(B)**. The PAM sequence is highlighted in blue. The frequency of detected off-targeting events in embryos **(C)**.

The independent off-targeting detection identified two locations in the genome of *RAG2/IL2RG* pigs that were suspected to be off-targeting events introduced by Cas9. Two genes, epithelial cell transforming 2 like (*ECT2L*) and DDRGK domain containing 1 (*DDRGK1*), were found to contain locus-specific modifications in at least one of the 20 embryos analyzed (Fig. 3). These modifications occur next to a potential PAM sequence and have not been shown in other pig breeds; therefore, the modifications were classified as true off-targeting events brought on by Cas9. Analysis of the *APP* knockout pigs did not detect any potential off-targeting events.

### Genome integrity in various tissues derived from genome-edited pigs

Multiple tissues from *APP* knockout pigs were used for WGS to identify any tissue-specific genome variation or targeting efficiency. No differences in the frequency of off-targeting were detected in the tissues. In fact, no noticeable off-targeting event was identified in the tissues, indicating high specificity of CRISPR-Cas system used to target *APP*. However, the use of WGS was able to detect mosaicism that can occur by injecting the CRISPR-Cas system in developing embryos in one of our GE pig models. From the *APP* knockout pigs, we analyzed genomic DNA from lung, brain, muscle, skin, spleen, and WBCs. Only pig #2–3 possessed a combination of four different alleles for the on-target locus in the brain, muscle, and spleen (Table 4). The lung tissue from pig #2–3 did not contain any modifications, and only wild-type *APP* sequence was identified. All other pigs contained the expected on-target modifications and were not mosaic in any other tissue.

**Table 4.** Identification of mosaicism in one *APP* genome edited pig

Pig	Tissue	Location	Sequence deletion/modifications	Resulting sequence
2–3	Brain	189715872	TCCAGGCGGCCAGCAGGACCAGTG	T
2–3	Brain	189715888	GA	G
2–3	Brain	189715890	CCAGTG	C
2–3	Brain	189715896	CCAAACCGGGCAGCATCGCGACCCTGCGCGGG	C
2–3	Lung		No variation	
2–3	Muscle	189715888	GA	G
2–3	Muscle	189715890	CCAGTG	C
2–3	Muscle	189715896	CCAAACCGGGCAGCATCGCGACCCTGCGCGGG	C
2–3	Spleen	189715872	TCCAGGCGGCCAGCAGGACCAGTG	T
2–3	Spleen	189715888	GA	G
2–3	Spleen	189715890	CCAGTG	C
2–3	Spleen	189715896	CCAAACCGGGCAGCATCGCGACCCTGCGCGGG	C

## Discussion

With the rise in use of the CRISPR-Cas system, interest in developing methods for detecting off-target sites associated with Cas9 is being increased. The possibility of producing unintended modifications in unknown regions of the genome is a major concern of using genome editing technology, especially with clinical application of CRISPR-Cas system to cure genetically linked diseases. Many sgRNA design tools, such as CRISPOR,^13^ predict the off-target possibilities of a given guide. However, the possibility of producing an unknown or unintended modification still exists. To further gain understanding of off-targeting events introduced by the CRISPR-Cas system, numerous *in vitro* methods have been developed to identify off-target sites in purified genomic DNA (SITE-Seq, Circle-SEQ, Digenome-Seq, among others).^7–9^ While these systems reflect cleavage of the genome by the designed CRISPR-Cas system, the system cannot calculate actual level of CRISPR-Cas system in target cell types or organisms. In fact, different cell types may have various genome editing efficiencies presumably due to different amounts of CRISPR-Cas to interact with the genome.^27^

Detection of off-targeting system is often based on monitoring changes in genomic sequences after genome editing.^28–31^ For instance, genome variances compared to a reference genome or following genome editing events can reveal on- and off-targeting events introduced due to genome editing systems. However, detection of off-targeting events in animal models with an unknown genetic background is difficult to determine because of existing natural variations to the reference genome. Unlike conventional rodent models, most pig breeds used in research have heterogeneous genetic backgrounds. In addition, during genetic engineering to establish novel pig models, unknown maternal genetic background is likely to be introduced because the oocyte source for producing GE embryos is normally abattoir-derived ovaries. Because of the unknown genetics, detection of off-targeting in GE pigs is extremely difficult. The reference genome of the pig, that is, *Sscrofa* 11.1, is also derived from a single pig with a specific genetic background and, therefore, may not capture genetic variation or changes caused by CRISPR-Cas system accurately.^32^ The heterogeneity in the genome may also create a challenge to generate a comprehensive list of potential off-targeting candidates using software such as Cas-OFFinder. However, because sgRNAs used in this study were designed to recognize exon sequences which are likely to be conserved amongst different breeds and we allowed for up to six base pair mismatches when generating the off-targeting list, we should have captured potential off-targeting candidates on a global level.

The high level of natural variation that exists among different pig breeds complicates the detection of true off-targeting events. Most of the off-targeting research published previously has been on mouse models derived from inbred mouse strains,^33^ homogeneous cell populations, or using genome mapping of background cell cultures.^34^ In this study, WGS was used to detect the level of off-targeting events on a global scale. The level of variation in all three gene-edited pig models compared to the pig reference genome was very similar, and even one of our paternal wild-type pig lines contained a high level of variation to the reference genome. This method of using WGS and Cas-OFFinder to find overlapping variants provided a means to rapidly identify and filter for variances that are likely to be off-targeting events. It is possible that the lack of parental genome information still hinders identification of all off-targeting events and labeling variations to be CRISPR-Cas origin. However, our ability to generate gene-edited embryos using the same sgRNAs and analyzing the resulting embryos validated effectiveness of the pipeline analysis and conclude some of variations to be introduced by CRISPR-Cas system. Our pipeline detected two novel off-targeting locations in our gene-edited pig models without having to install any complicated coding to dissect off-targeting events versus natural variation. Since technology to perform whole genome sequencing in blastocysts is available,^35^ our pipeline can be used to identify the realistic level of off-targeting from designed CRISPR-Cas systems. This would allow the selection of the safest CRISPR-Cas system prior to performing embryo transfers, which is a significant investment in large animal species such as pigs.

The number of total variations in our gene-edited and wild-type control pigs indicate the heterogenous nature of the pig genome and underscore the safety of the CRISPR-Cas system, that is, no noticeable genome instability at the global level. The WGS was performed using Illumina NovaSeq based sequencing, and the genome was assembled from 150 bp reads. The sequencing reads were mapped to the pig reference sequence. While the genome assembly was successfully used to detect overall variances in the genome, using short reads has some limitations as gene inversions or certain regions on chromosomes may have not been captured from the approach. Combining our strategy with a longer read application such as PacBio or Nanopore sequencing^36,37^ may give a more accurate assessment of off-targeting events and impact of genome editing on genome integrity.

The level of off-targeting events identified in the gene-edited pigs in this study was low. Most pigs carried no detectable off-targeting events in the genome, and independent validation of those off-targeting events in newly generated gene-edited embryos revealed that off-targeting frequency was low; only two out of eight off-targeting locations were confirmed. The detection rate may be increased if more gene-edited pigs/embryos were screened; however, the low frequency emphasizes that these off-targeted sequences are not likely to be carried to next generation after breeding and phenotypes observed in founder gene-edited pigs and its progeny should be rooted from the on-target modification.

Mosaicism has been identified as a potential problem brought on from the direct injection of the CRISPR/Cas system into oocytes or zygotes.^38^ In this study, we show that only one *APP* knockout pig derived from oocyte injections, out of three pigs, was mosaic. The other two pigs had on-target modifications likely introduced prior to the first embryo cleavage event and, therefore, did not possess more than two alleles. Furthermore, we did not detect any tissue specific bias in genome editing or off-targeting events in this pig model. However, it is difficult to draw more conclusions with the current sensitivity as no off-targeting events were detected in these *APP* knockout pigs because of the low sample number used in this study.

## Conclusions

The method presented here offers a simplified approach to detect off-targeting events in an unknown genetic background. Using this method, we were able to identify on-target modifications, mosaic tissues in GE pigs, known off-targeting modifications, as well as novel off-targeting events. Pigs are considered a suitable preclinical animal model,^39^ and the ability to monitor off-targeting events following application of genome editing tools will increase their value as a preclinical model when genome editing technology is already entering the clinic.^40,41^ Our finding will offer a guideline to detect off-targeting events in pigs for biomedical applications and imply the level of off-targeting events that could be encountered while applying genome editing technology in the clinics.

## Data Availability

All data generated during this study are published within this article and its supplementary information file. All other data are available from the corresponding author upon reasonable request. Raw files are available in the NCBI Sequence Read Archive (SRA) repository—BioProject: **PRJNA1024571.** The BioProject and associated SRA metadata are available at https://gcc02.safelinks.protection.outlook.com/?url=https%3A%2F%2Fdataview.ncbi.nlm.nih.gov%2Fobject%2FPRJNA1024571%3Freviewer%3Dv3krpjojr4po5bqmd36a1vddnn&data=05%7C01%7Cbethany.redel%40usda.gov%7C3feb0b1d949c4785580708dbcb547270%7Ced5b36e701ee4ebc867ee03cfa0d4697%7C1%7C0%7C638327335787035177%7CUnknown%7CTWFpbGZsb3d8eyJWIjoiMC4wLjAwMDAiLCJQIjoiV2luMzIiLCJBTiI6Ik1haWwiLCJXVCI6Mn0%3D%7C3000%7C%7C%7C&sdata=jgVVzaJ5pXlTwoBHOkRymPi3vn4C82RHry2RRLCkWKA%3D&reserved=0 for reviewer access.

## List of abbreviations

AR: Androgen receptor
RBFOX1: RNA binding fox-1 homolog 1
ECT2L: Epithelial cell transforming 2 like
DDRGK1: DDRGK domain containing 1
APP: Amyloid beta precursor protein
CRISPR: Clustered, regularly interspaced, short palindromic repeat
GE: Gene-edited
Cas9: CRISPR associated protein 9
sgRNA: Single guide ribonucleic acid
PAM: Protospacer Adjacent Motif
DSB: Double stranded break
InDels: Insertion-Deletions
WGS: Whole genome sequencing
IGH: Immunoglobulin heavy locus
RPS6KC1: Ribosomal protein S6 kinase C1
BLAST: Basic local alignment search tool
WBCs: White blood cells
SNPs: Single nucleotide polymorphisms

## References

[1] Cong L, Ran FA, Cox D, et al. Multiplex genome engineering using CRISPR/Cas systems. Science 2013;339(6121):819–823; doi: 10.1126/science.1231143 23287718 PMC3795411

[2] Jinek M, Chylinski K, Fonfara I, et al. A programmable dual-RNA-guided DNA endonuclease in adaptive bacterial immunity. Science 2012;337(6096):816–821; doi: 10.1126/science.1225829 22745249 PMC6286148

[3] Guo X, Li X-J. Targeted genome editing in primate embryos. Cell Res 2015;25(7):767–768; doi: 10.1038/cr.2015.64 26032266 PMC4493275

[4] Lunney JK, Van Goor A, Walker KE, et al. Importance of the pig as a human biomedical model. Sci Transl Med 2021;13(621):eabd5758; doi: 10.1126/scitranslmed.abd5758 34818055

[5] OECD. Food, Nations AOotU. OECD-FAO Agricultural Outlook 2023-2032. 2023.

[6] Whitworth KM, Green JA, Redel BK, et al. Improvements in pig agriculture through gene editing. CABI Agric Biosci 2022;3(1):41; doi: 10.1186/s43170-022-00111-9 35755158 PMC9209828

[7] Cameron P, Fuller CK, Donohoue PD, et al. Mapping the genomic landscape of CRISPR–Cas9 cleavage. Nat Methods 2017;14(6):600–606; doi: 10.1038/nmeth.4284 28459459

[8] Tsai SQ, Nguyen NT, Malagon-Lopez J, et al. CIRCLE-seq: A highly sensitive in vitro screen for genome-wide CRISPR–Cas9 nuclease off-targets. Nat Methods 2017;14(6):607–614; doi: 10.1038/nmeth.4278 28459458 PMC5924695

[9] Kim D, Bae S, Park J, et al. Digenome-seq: Genome-wide profiling of CRISPR-Cas9 off-target effects in human cells. Nat Methods 2015;12(3):237–243, 1 p following 243; doi: 10.1038/nmeth.3284 25664545

[10] Carey K, Ryu J, Uh K, et al. Frequency of off-targeting in genome edited pigs produced via direct injection of the CRISPR/Cas9 system into developing embryos. BMC Biotechnol 2019;19(1):25; doi: 10.1186/s12896-019-0517-7 31060546 PMC6501304

[11] Yugo DM, Heffron CL, Ryu J, et al. Infection dynamics of hepatitis E Virus in wild-type and immunoglobulin heavy chain knockout J(H)(-/-) Gnotobiotic Piglets. J Virol 2018;92(21); doi: 10.1128/jvi.01208-18 PMC6189505 30111571

[12] Ryu J, Prather RS, Lee K. Use of gene-editing technology to introduce targeted modifications in pigs. J Anim Sci Biotechnol 2018;9:5; doi: 10.1186/s40104-017-0228-7 29423214 PMC5787920

[13] Concordet J-P, Haeussler M. CRISPOR: Intuitive guide selection for CRISPR/Cas9 genome editing experiments and screens. Nucleic Acids Res 2018;46(W1):W242–W245; doi: 10.1093/nar/gky354 29762716 PMC6030908

[14] Lei S, Ryu J, Wen K, et al. Increased and prolonged human norovirus infection in RAG2/IL2RG deficient gnotobiotic pigs with severe combined immunodeficiency. Sci Rep 2016;6:25222; doi: 10.1038/srep25222 27118081 PMC4846862

[15] Redel BK, Spate LD, Prather RS. In vitro maturation, fertilization, and culture of pig oocytes and embryos. Methods Mol Biol 2019;2006:93–103; doi: 10.1007/978-1-4939-9566-0_6 31230274

[16] Yoshioka K, Suzuki C, Tanaka A, et al. Birth of piglets derived from porcine zygotes cultured in a chemically defined medium. Biol Reprod 2002;66(1):112–119. 11751272 10.1095/biolreprod66.1.112

[17] Redel BK, Tessanne KJ, Spate LD, et al. Arginine increases development of in vitro-produced porcine embryos and affects the protein arginine methyltransferase?dimethylarginine dimethylaminohydrolase?nitric oxide axis. Reprod Fertil Dev 2015;27(4):655–666; doi: 10.1071/RD14293 25765074 PMC4726483

[18] Chen PR, Redel BK, Spate LD, et al. Glutamine supplementation enhances development of in vitro-produced porcine embryos and increases leucine consumption from the medium. Biol Reprod 2018;99(5):938–948; doi: 10.1093/biolre/ioy129 29860318 PMC6297286

[19] Di Tommaso P, Chatzou M, Floden EW, et al. Nextflow enables reproducible computational workflows. Nat Biotechnol 2017;35(4):316–319; doi: 10.1038/nbt.3820 28398311

[20] Buckley RM, Davis BW, Brashear WA, et al. A new domestic cat genome assembly based on long sequence reads empowers feline genomic medicine and identifies a novel gene for dwarfism. PLoS Genet 2020;16(10):e1008926; doi: 10.1371/journal.pgen.1008926 33090996 PMC7581003

[21] Li H. Minimap2: Pairwise alignment for nucleotide sequences. Bioinformatics 2018;34(18):3094–3100; doi: 10.1093/bioinformatics/bty191 29750242 PMC6137996

[22] Poplin R, Ruano-Rubio V, DePristo MA, et al. Scaling accurate genetic variant discovery to tens of thousands of samples. bioRxiv 2018;201178; doi: 10.1101/201178

[23] DePristo MA, Banks E, Poplin R, et al. A framework for variation discovery and genotyping using next-generation DNA sequencing data. Nat Genet 2011;43(5):491–498; doi: 10.1038/ng.806 21478889 PMC3083463

[24] Bae S, Park J, Kim J-S. Cas-OFFinder: A fast and versatile algorithm that searches for potential off-target sites of Cas9 RNA-guided endonucleases. Bioinformatics 2014;30(10):1473–1475; doi: 10.1093/bioinformatics/btu048 24463181 PMC4016707

[25] Groenen MAM, Archibald AL, Uenishi H, et al. Analyses of pig genomes provide insight into porcine demography and evolution. Nature 2012;491(7424):393–398; doi: 10.1038/nature11622 23151582 PMC3566564

[26] Warr A, Affara N, Aken B, et al. An improved pig reference genome sequence to enable pig genetics and genomics research. Gigascience 2020;9(6); doi: 10.1093/gigascience/giaa051 PMC7448572 32543654

[27] Shen MW, Arbab M, Hsu JY, et al. Predictable and precise template-free CRISPR editing of pathogenic variants. Nature 2018;563(7733):646–651; doi: 10.1038/s41586-018-0686-x 30405244 PMC6517069

[28] Schaefer KA, Wu WH, Colgan DF, et al. Unexpected mutations after CRISPR-Cas9 editing in vivo. Nat Methods 2017;14(6):547–548; doi: 10.1038/nmeth.4293 28557981 PMC5796662

[29] Frock RL, Hu J, Meyers RM, et al. Genome-wide detection of DNA double-stranded breaks induced by engineered nucleases. Nat Biotechnol 2015;33(2):179–186; doi: 10.1038/nbt.3101 25503383 PMC4320661

[30] Smith C, Gore A, Yan W, et al. Whole-genome sequencing analysis reveals high specificity of CRISPR/Cas9 and TALEN-Based genome editing in human iPSCs. Cell Stem Cell 2014;15(1):12–13; doi: 10.1016/j.stem.2014.06.011 24996165 PMC4338993

[31] Iyer V, Shen B, Zhang W, et al. Off-target mutations are rare in Cas9-modified mice. Nat Methods 2015;12(6):479–479; doi: 10.1038/nmeth.3408 26020497

[32] Johnsson M, Hickey JM, Jungnickel MK. Building in vitro tools for livestock genomics: Chromosomal variation within the PK15 cell line. BMC Genomics 2024;25(1):49; doi: 10.1186/s12864-023-09931-z 38200430 PMC10782621

[33] Peterson KA, Khalouei S, Hanafi N, et al. Whole genome analysis for 163 gRNAs in Cas9-edited mice reveals minimal off-target activity. Commun Biol 2023;6(1):626; doi: 10.1038/s42003-023-04974-0 37301944 PMC10257658

[34] Tsai HH, Kao HJ, Kuo MW, et al. Whole genomic analysis reveals atypical non-homologous off-target large structural variants induced by CRISPR-Cas9-mediated genome editing. Nat Commun 2023;14(1):5183; doi: 10.1038/s41467-023-40901-x 37626063 PMC10457329

[35] Peters BA, Kermani BG, Alferov O, et al. Detection and phasing of single base de novo mutations in biopsies from human in vitro fertilized embryos by advanced whole-genome sequencing. Genome Res 2015;25(3):426–434; doi: 10.1101/gr.181255.114 25672852 PMC4352880

[36] Rhoads A, Au KF. PacBio sequencing and its applications. Genomics Proteomics Bioinformatics 2015;13(5):278–289; doi: 10.1016/j.gpb.2015.08.002 26542840 PMC4678779

[37] Jain M, Olsen HE, Paten B, et al. The oxford nanopore MinION: Delivery of nanopore sequencing to the genomics community. Genome Biol 2016;17(1):239; doi: 10.1186/s13059-016-1103-0 27887629 PMC5124260

[38] Vilarino M, Suchy FP, Rashid ST, et al. Mosaicism diminishes the value of pre-implantation embryo biopsies for detecting CRISPR/Cas9 induced mutations in sheep. Transgenic Res 2018;27(6):525–537; doi: 10.1007/s11248-018-0094-x 30284144

[39] Redel BK, Prather RS. Meganucleases revolutionize the production of genetically engineered pigs for the study of human diseases. Toxicol Pathol 2016;44(3):428–433; doi: 10.1177/0192623315613160 26516165 PMC4805444

[40] Khirallah J, Eimbinder M, Li Y, et al. Clinical progress in genome-editing technology and in vivo delivery techniques. Trends Genet 2023;39(3):208–216; doi: 10.1016/j.tig.2022.12.001 36669950 PMC9974761

[41] Gillmore JD, Gane E, Taubel J, et al. CRISPR-Cas9 In vivo gene editing for transthyretin amyloidosis. N Engl J Med 2021;385(6):493–502; doi: 10.1056/NEJMoa2107454 34215024

